# Self-reported hearing difficulties and changes in life-space mobility among community-dwelling older adults: a Two-year follow-Up study

**DOI:** 10.1186/s12877-015-0119-8

**Published:** 2015-10-12

**Authors:** Hannele Polku, Tuija M. Mikkola, Merja Rantakokko, Erja Portegijs, Timo Törmäkangas, Taina Rantanen, Anne Viljanen

**Affiliations:** Gerontology Research Center and Department of Health Sciences, University of Jyväskylä, P.O. Box 35, FI-40014 Jyväskylä, Finland

**Keywords:** Hearing, Life-space, Aging, Cohort, Longitudinal study

## Abstract

**Background:**

Life-space mobility reflects individuals’ actual mobility and engagement with society. Difficulty in hearing is common among older adults and can complicate participation in everyday activities, thus restricting life-space mobility. The aim of this study was to examine whether self-reported hearing predicts changes in life-space mobility among older adults.

**Methods:**

We conducted a prospective cohort study of community-dwelling older adults aged 75–90 years (*n* = 848). At-home face-to-face interviews at baseline and telephone follow-up were used. Participants responded to standardized questions on perceived hearing at baseline. Life-space mobility (the University of Alabama at Birmingham Life-Space Assessment, LSA, range 0–120) was assessed at baseline and one and two years thereafter. Generalized estimating equations were used to analyze the effect of hearing difficulties on changes in LSA scores.

**Results:**

At baseline, participants with major hearing difficulties had a significantly lower life-space mobility score than those without hearing difficulties (mean 54, 95 % CI 50–58 vs. 57, 95 % CI 53–61, *p* = .040). Over the 2-year follow-up, the life-space mobility score declined in all hearing categories in a similar rate (main effect of time *p* < .001, group x time *p* = .164). Participants with mild or major hearing difficulties at baseline had significantly higher odds for restricted life-space (LSA score < 60) at two years (OR 1.8, 95 % CI 1.0–3.2 and 2.0, 95 % CI 1.0–3.9, respectively) compared to those without hearing difficulties. The analyses were adjusted for chronic conditions, age, sex and cognitive functioning.

**Conclusions:**

People with major hearing difficulties had lower life-space mobility scores at baseline but did not exhibit accelerated decline over the follow-up compared to those without hearing difficulties. Life-space mobility describes older people’s possibilities for participating in out-of-home activities and access to community amenities, which are important building blocks of quality of life in old age. Early recognition of hearing difficulties may help prevent life-space restriction.

## Background

Difficulty in hearing is common among older adults [1]. The prevalence of hearing difficulties rises markedly with increasing age [2–4], affecting about two-thirds of people aged 70 years [5] and 90 % of adults aged 80 or above [2]. Most often older adults’ hearing difficulties result from degenerative changes in cochlear structure and the auditory pathway [6], although other factors such as exposure to noise, ototoxic drugs and a number of medical conditions, such as diabetes, may induce deterioration in hearing [7].

Previous studies have suggested that hearing difficulties can complicate engagement in everyday life situations; especially those requiring communication with other people [8, 9]. Difficulty in following conversations is one of the most common worries among older adults [10], especially in challenging listening conditions such as in the presence of background noise or sound-reverberating environments [11]. It has also been shown that older people with hearing difficulty experience more walking difficulties [12, 13], poorer postural balance, higher risk for falls [14] and fear of falling [15], than those without hearing impairment, factors which may also compromise possibilities for participation in everyday activities.

Life-space mobility reflects the size of the spatial area a person moves through in daily life, the frequency of moving and the need for assistance [16, 17]. While the assessment of mobility limitations or disabilities reflect a person’s potential capacity to perform the particular activities [18, 19], it does not reflect actual participation in activities in question [20, 21]. Life-space mobility, in turn, reflects what people *actually* do, as it describes total mobility, thereby providing us with a broad picture of a person’s engagement with the community [16, 20, 22, 23]. Life-space mobility is a measure of the balance between an individual’s resources (e.g. physiological and psychological capacity) and the demands of the environment in the context of a person’s real-life situation [16, 19].

We are aware of only one earlier study that has investigated the association between hearing problems and life-space mobility. After adjustment for potential confounders, Allman et al. found that hearing difficulty did not predict decline in life-space mobility in a 18-month follow-up [24].

The aim of our study was to examine whether self-reported hearing is associated cross-sectionally with life-space mobility among 75 to 90-year-old community-dwelling older adults and, whether self-reported hearing difficulties at baseline predict changes in life-space mobility at follow-up one and two years later.

## Methods

### Study design and participants

This study is part of the “Life-space mobility in old age” (LISPE) project, which is a prospective cohort study of community-dwelling older adults. A more detailed description of the study design has been published earlier [25]. Briefly, for this study, a random sample of 2550 community-dwelling 75 to 90-year-old residents of the Finnish municipalities of Jyväskylä and Muurame was drawn from the national population register. Individuals were contacted by letter and over the phone to enquire about their willingness, and assess their suitability, to take part in the study. The inclusion criteria were community-dwelling in the study area, and able to communicate. After screening, a total of 848 eligible people agreed to participate and were interviewed in their homes during spring 2012. Of them, 816 participated in the one-year follow-up and 761 participated in the two-year follow-up. During the two-year period, 41 participants died, 15 moved into institutional care, and 12 were excluded due to loss of the ability to communicate. Other reasons for attrition were moving outside the study area (*n* = 6), poor health (*n* = 5), not willing to continue (*n* = 6), and not reached (*n* = 2). The LISPE project was approved by the Ethical Committee of the University of Jyväskylä. Participants were informed about the project and signed a written informed consent.

### Measurements

#### Hearing

Hearing at baseline was assessed by asking the following three questions:“ Do you have difficulties hearing when having a conversation with several people simultaneously?”, “Do you have difficulty hearing when conversing with another person in the presence of noise?”, and “Do you have difficulties hearing where a particular sound (i.e. phone ringing, sound of a car) is coming from?”. The participants were asked to estimate their level of difficulty when using a hearing aid if they had one. The response categories were 1) No difficulty (0 points), 2) Sometimes, some difficulty (1 point), and 3) Yes, major difficulty (2 points). Scores were summed and the resulting scale was divided into three categories: no hearing difficulties (score 0), mild hearing difficulties (score 1–2), and major hearing difficulties (3 or higher). The reason for this categorization was that having major hearing difficulties should involve some difficulty in all three situations or major difficulty in at least one situation plus some difficulty in another situation [26].

#### Life-space mobility

Life-space mobility was measured using the University of Alabama at Birmingham Life-Space Assessment (LSA) questionnaire [16] at baseline and in both follow-ups. The LSA is based on self-report and comprises 15 items measuring mobility through different life-space levels (bedroom, other rooms in the home, outside home, neighborhood, town, beyond town) in terms of distance, frequency and independence during the 4 weeks preceding the assessment. In this study we used the life-space mobility score [16] ranging from 0 to 120 (higher scores indicate better life-space mobility). A score of <60 on the life-space assessment represents restricted life-space [18, 23]. The reliability and validity of the questionnaire has been found to be acceptable [16, 20, 27].

#### Potential confounders

Factors previously found to be potential risk factors for life-space mobility decline and hearing difficulties were considered potential confounders [7, 13, 16, 22, 28–31]. *Basic demographic and socioeconomic indicators* of the study subjects included age, sex and number of years of education. *Self-reported physician diagnosed chronic conditions* were obtained from a list of 22 chronic conditions and with an open-ended question. Chronic conditions that could theoretically be linked to hearing difficulties and life-space mobility, namely diabetes, rheumatoid arthritis, cardiac, circulatory and neurological diseases were chosen as potential covariates. *Cognitive functioning* was assessed using the Mini- Mental State Examination (MMSE) [32].

### Statistical analyses

The Kruskal-Wallis test was used for continuous variables and chi-square tests for categorical variables to compare the baseline characteristics between the hearing groups. Characteristics of the participants are described using medians and interquartile ranges (IQR) or percentages. In the two-year follow-up, generalized estimating equations (GEE) models were used to test the significance of the association of self-reported hearing difficulties on changes in life-space mobility over time. In addition, logistic regression models were used to investigate whether self-reported hearing difficulties at baseline were associated with higher odds for life-space restriction at baseline and at the second follow-up. In the logistic regression analyses, the life-space mobility score was dichotomized by using a cut-off score of 60. Odds ratios for life-space restriction at the second follow-up were calculated only for those participants who had unrestricted life-space at baseline. Age and sex were included in all models. In addition, cognitive functioning, diabetes and cardiac, circulatory, and neurological diseases had also a significant effect (*p* < .05) on the parameters and were used as covariates in the adjusted models. Education did not have a significant effect in the model and was therefore excluded from the final model.

Life-space mobility data were available for all 848 participants at baseline, 806 participants at the one-year follow-up and 757 participants at the two-year follow up. Data for hearing difficulties at baseline were available for 844 participants. As hearing data were missing for less than 1 % of the participants, missing values were not imputed.

Parameter estimates for the GEE models [33] were obtained from IBM SPSS Statistics for Windows software, version 22.0.0.1. Confidence intervals for the estimated marginal means were computed in the R programming environment, version 3.1.1.

## Results

### Baseline results

The median age of all the participants (*n* = 848) was 80.0 years at baseline (interquartile range 8.0, mean 80, SD 4.3) and 62 % of the participants were women. At baseline, the median life-space mobility score in the total sample was 64.0 (IQR 30.4, mean 63.9, SD 20.6), ranging from 8 to 120. In the total sample, 33 % reported no hearing difficulties, 45 % reported mild hearing difficulties and 22 % reported major hearing difficulties, while having a hearing aid was reported by 13.5 %. Baseline characteristics of the participants categorized according to self-reported difficulty in hearing are presented in Table 1. There were no differences in distribution of hearing difficulties at the baseline between those participants who were followed and those who were lost during the follow-up.

**Table 1:** Baseline characteristics of the participants categorized according to self-reported difficulty in hearing

	No hearing difficulties (*n* = 276)		Mild hearing difficulties (*n* = 381)		Major hearing difficulties (*n* = 187)		
	Median	IQR	Median	IQR	Median	IQR	p^a^
Age	79.0	6.0	80.0	8.0	81.0	8.0	<.001
Education in years	9.0	5.0	9.0	6.0	8.0	5.0	.224
MMSE score	27.0	3.0	27.0	3.0	26.0	4.0	<.001
	%	(n)	%	(n)	%	(n)	p^b^
Women	63.4	175	59.3	226	65.2	122	.328
Hearing aid owner	1.5	4	10.0	38	38.2	71	<.001
Cardiac diseases	31.9	88	43.6	166	54.5	102	<.001
Circulatory diseases	58.0	160	69.6	265	68.4	128	.005
Diabetes	15.9	44	17.8	68	19.8	37	.562
Neurological diseases	6.9	19	6.8	26	8.0	15	.859
Rheumatoid arthritis	4.0	11	5.0	19	8.0	15	.153

^a^Kruskal-Wallis H-test

^b^Chi-Square test

Participants with major hearing difficulties had a significantly lower life-space mobility score at baseline (mean 62, 95 % CI 59–64) compared to participants without hearing difficulties (68, 95 % CI 65–70, *p* < .001) or with mild hearing difficulties (65, 95 % CI 64–67, *p* = .022). Participants with mild hearing difficulties did not statistically significantly differ from those without hearing difficulties in their life-space mobility score (*p* = .141). After further adjustment for cognitive functioning (MMSE), diabetes, cardiac, circulatory and neurological diseases and rheumatoid arthritis, the statistically significant difference in life-space mobility score between persons with mild hearing difficulty and those with major hearing difficulty became non-significant (56, 95 % CI 53–60 vs. 54, 95 % CI 50–58, *p* = .106), while between participants without hearing difficulties (57, 95 % CI 53–61) and those with major hearing difficulty the difference remained significant (*p* = .040).

At baseline, participants with mild hearing difficulty had 1.5 (95 % CI 1.0-2.1), and those with major hearing difficulty 2.1(95 % CI 1.4–3.2) times higher odds for restricted life-space compared to those without hearing difficulties. After further adjustment for the covariates, the participants with mild hearing difficulty no longer differed significantly from those without hearing difficulties (Table 2).

**Table 2:** The odds for restricted life-space by categories of self-reported hearing difficulties at baseline and odds for incident of life-space restriction at second follow-up

		Baseline (n = 844)						
			Model 1^a^			Model 2^b^		
Baseline hearing difficulties	n	Life-space restriction %	OR	95 % CI	p	OR	95 % CI	p
No hearing difficulties	276	30.9	1			1		
Mild hearing difficulty	381	41.2	1.5	1.0-2.1	.033	1.4	0.9-2.0	.107
Major hearing difficulty	187	54.5	2.1	1.4-3.2	<.001	1.8	1.2-2.8	.007
		Two-year follow-up (n = 465)						
			Model 1^a^			Model 2^b^		
	n	Life-space restriction %	OR	95 % CI	p	OR	95 % CI	p
No hearing difficulties	177	16.4	1			1		
Mild hearing difficulty	210	27.1	1.8	1.1-3.1	.025	1.8	1.0-3.2	.035
Major hearing difficulty	78	29.5	2.1	1.1-4.0	.031	2.0	1.0-3.9	.057

Group without hearing difficulties at baseline is the reference group. Odds ratios for restricted life-space (=life-space mobility score <60) during the two-year follow-up were calculated only for those participants who had unrestricted life-space at baseline

^a^adjusted for sex and age

^b^adjusted for sex, age, cognitive functioning (MMSE),rheumatoid arthritis, diabetes, cardiac, circulatory and neurological diseases

### Follow-up results

At the two-year follow-up the median life-space mobility score of all the participants was 63 (mean 61.4, SD 22.1, IQR 35).

**Fig. 1:**
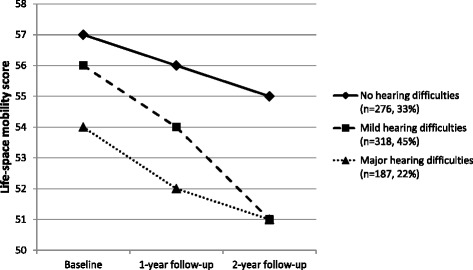
Estimated marginal means for life-space mobility by categories of self-reported hearing over a two-year follow-up. Fully adjusted model

Over the 2-year follow-up, the life-space mobility score declined in all the hearing categories (main effect of time *p* < .001) and at a similar rate (group x time *p* = .164) (Fig. 1). Hearing had a significant effect on the life-space mobility score over the two-year follow-up (main effect of group *p* = .049). During the two-year follow-up, the difference in life-space mobility score remained significant (*p* = .001) between the participants without hearing difficulties and those with major hearing difficulties. At the end of the follow-up, participants without hearing difficulties had a higher life-space mobility score (Mean 65, 95 % CI 63–68) than those with either mild (60, 95 % CI 58–62, *p* = .002) or major hearing difficulties (59, 95 % CI 56–62, *p* = .001). Participants with mild hearing difficulties and those with major hearing difficulties did not differ in their life-space mobility score at two-years (*p* = .544). Further adjustments for cognitive functioning, rheumatoid arthritis, diabetes, cardiac, circulatory or neurological diseases did not essentially change the differences between hearing categories, as life-space mobility score remained significantly higher among participants without hearing difficulties (55, 95 % CI 51–59) compared to those with mild (51, 95 % CI 47–55, *p* = .012) or major (51, 95 % CI 47–55, *p* = .037) hearing difficulties.

Among the participants with unrestricted life-space at baseline (LSA-score > 60, *n* = 465), those with mild or major hearing difficulties at baseline had significantly higher odds for life-space restriction at the second follow-up compared to those without hearing difficulties. While further adjustments did not materially change these results, the odds ratio became borderline significant (*p* = .057) for those with major hearing difficulty (Table 2).

## Discussion

Our results showed that hearing difficulties were associated with poorer life-space mobility in community-dwelling older adults. Life-space mobility declined steadily in all the hearing categories over the two-year follow-up period. However, persons who did not have hearing difficulties at baseline had significantly higher life-space mobility score both at baseline and at the two-year follow up compared to those who reported hearing difficulties, even when controlling for the presence of chronic medical conditions and the influence of cognitive functioning, age and sex. It is likely that this trend existed before the cohort was followed up. Thus, although the changes observed in the life-space mobility score between the hearing categories were modest, the results indicate that hearing problems may contribute to restrictions in life-space mobility over time among older adults.

According to previous studies [18, 23], a score of 60 and higher on the life-space assessment represents unrestricted life-space, defined as a “person’s ability to get out of one’s neighborhood independently” and thus is a marker of independent mobility and resilient aging [20]. Our findings suggest that older adults with hearing difficulties reach this critical threshold for restricted life-space mobility sooner than older adults without hearing difficulties.

To our knowledge, the 18-month follow-up study of Allman and colleagues [24] is the only previous study to examine the association between hearing problems and life-space mobility. In their study, the correlation between hearing difficulties and decline in life-space mobility was attenuated when adjusted for other health conditions. It is possible that their study lacked the power needed to observe an association, as their sample contained significantly fewer people who were categorized as having hearing problems.

There may be several explanations for our findings. According to earlier studies, communication problems are the most prevalent participation restrictions mentioned by older adults with hearing difficulties [8]. For older adults, the desire to interact with other people is typically one of the main reasons for going outside the home [34]. However, challenges in communication may cause feelings of frustration, embarrassment and being left out of things, which in turn may lead to social withdrawal [10, 26, 35–37] and reduce participation in social activities [9, 13], thereby reducing life-space mobility. Older adults with a higher frequency of social participation and greater number of social networks are more likely to have larger life space than those with less social contacts [22, 38]. As life-space mobility reflects individuals’ actual mobility and frequency of participation in activities outside the home, it may be that poorer life-space mobility is indicative of a decreased desire among older persons with hearing difficulties to be active and exploit community amenities and engage in social activities. Thus changes in life-space mobility can also reflect the adaptations [18] older adults make in response to impaired hearing. For example, a person may not withdraw completely from situations that pose a challenge to hearing, but engage in them less often [9].

Hearing difficulties not only impede communication with other people, but may also impair the ability to observe environmental hazards. Acoustic information supports observation of the environment while moving [39], and hence its reception is important, e.g., in preparing for elements of danger such as motor vehicles. Hearing difficulties compromise the ability to localize sounds reliably [6, 10], which may make it difficult to piece together what to monitor in the environment, leading eventually to reluctance to expose oneself to such challenging situations and thus reduced activity outside the home. Hearing difficulties have also been reported to be associated with higher rates of walking difficulties [35] and decreased walking speed and postural balance as well as mobility decline and falls [12–14]. Walking and balance difficulties together with inaccurate environmental acoustic information may further impair safe mobility and reduce the desire of older adults to go outdoors, resulting in reduced life-space mobility. Avoidance of challenging acoustic environments may lead to a detrimental cycle where restrictions on life-space mobility cause further decline in physical ability and social relationships.

This study included a large population-based sample of community-dwelling older adults and there were very few missing data in the sample. Although the participants were rather well-functioning, the sample also included people with health problems [25]. It is thus reasonable to assume that the associations found here most likely represent those prevalent among the general population of comparable age. We used multiple questions to assess hearing difficulties in situations that are typically challenging for persons with age-related hearing impairment and we also asked the participants to evaluate the perceived degree of their hearing difficulties. This approach may yield a more comprehensive picture of the extent of hearing difficulties than that gained by using a single question.

A potential limitation of our study is that hearing was self-reported. However, self-reports of hearing are commonly used in epidemiological studies, and previous studies support the validity of self-reported measures of hearing impairment [26, 28, 35, 40]. Furthermore, self-reports are relevant since they make use of information about the difficulties older adults perceive in their everyday situations [41]. A short follow-up is another potential limitation of our study. All of our participants were at least 75 years old at baseline. As hearing decline is usually a gradual process, it is likely that the influence of hearing difficulties on life-space mobility were already present before the cohort was initiated. However, the age range between 75 and 90, of our participants corresponds to that when people are increasingly vulnerable to decline in life-space mobility due underlying changes in health, and in sensory and physical functions [24, 42]. Our results did not show a more accelerated decline in life-space mobility among those with hearing difficulties; however, the logistic regression with restricted life-space mobility as the outcome suggests that hearing difficulties precedes restrictions in life-space mobility. Although we may not have definitely established causation, it is unlikely that limitations in life-space mobility lead to self-reported hearing difficulties. However, we cannot exclude the possibility of residual confounding to the results caused by unmeasured factors.

Further studies with longer follow-ups starting from middle-age are needed to confirm the associations reported in this study. Also, some persons were excluded from the study because they were not able to communicate due to hearing problems during the initial telephone screening or home interview. Therefore, it is likely that the number of persons with severe hearing impairment in this study was under-represented. The associations observed between hearing and life-space mobility might have been stronger had these persons participated.

## Conclusions

To conclude, the present study provides new information on longitudinal changes in life-space mobility among older people with and without hearing difficulties. Given the increasing proportion of older adults in the population, it is particularly important to understand the role of hearing difficulties as a risk factor for restricted life-space mobility and consequent decreased participation in society. Our findings emphasize the need for early assessment and recognition of hearing difficulties in order to diminish the likelihood of subsequent losses in functional capacity. In future, additional attention should also be given to the balance between individual resources and environmental demands in older adults who have hearing difficulties. More specifically, it would be important to know more precisely how the acoustic characteristics of the environment and environmental noise affect older persons’ possibilities to maintain social relationships and active participation in daily life in real world situations.

## Abbreviations

LSA: the University of Alabama at Birmingham Life-Space Assessment (LSA) questionnaire
SE: Standard error
OR: Odds ratio
95 % CI: 95 % confidence interval
LISPE: “Life-space mobility in old age” study
MMSE: Mini-mental state examination
IQR: Interquartile range
GEE: Generalized estimating equations
